# Repulsive bimodal atomic force microscopy on polymers

**DOI:** 10.3762/bjnano.3.52

**Published:** 2012-06-20

**Authors:** Alexander M Gigler, Christian Dietz, Maximilian Baumann, Nicolás F Martinez, Ricardo García, Robert W Stark

**Affiliations:** 1 Center for NanoScience (CeNS) and Department of Earth and Environmental Sciences, Ludwig-Maximilians-Universität München, Theresienstraße 41, 80333 Munich, Germany; 2 Center of Smart Interfaces and Department of Materials Sciences, Technische Universität Darmstadt, Petersenstr. 32, 64287 Darmstadt, Germany; 3 Instituto de Microelectrónica de Madrid, c/ Isaac Newton 8, Tres Cantos, 28760 Madrid, Spain

**Keywords:** bimodal AFM imaging, diblock copolymer, polybutadiene, polystyrene

## Abstract

Bimodal atomic force microscopy can provide high-resolution images of polymers. In the bimodal operation mode, two eigenmodes of the cantilever are driven simultaneously. When examining polymers, an effective mechanical contact is often required between the tip and the sample to obtain compositional contrast, so particular emphasis was placed on the repulsive regime of dynamic force microscopy. We thus investigated bimodal imaging on a polystyrene-*block*-polybutadiene diblock copolymer surface and on polystyrene. The attractive operation regime was only stable when the amplitude of the second eigenmode was kept small compared to the amplitude of the fundamental mode. To clarify the influence of the higher eigenmode oscillation on the image quality, the amplitude ratio of both modes was systematically varied. Fourier analysis of the time series recorded during imaging showed frequency mixing. However, these spurious signals were at least two orders of magnitude smaller than the first two fundamental eigenmodes. Thus, repulsive bimodal imaging of polymer surfaces yields a good signal quality for amplitude ratios smaller than *A*_01_*/A*_02_ = 10:1 without affecting the topography feedback.

## Introduction

The compositional mapping of heterogeneous surfaces at nanometer resolution is one of the most common applications of atomic force microscopy. Resonant modes such as amplitude-modulated atomic force microscopy allow one to routinely image very delicate samples without introducing sample distortions [1–5]. In recent years, various multifrequency approaches for image-contrast enhancement in air and liquid environments have been established [6–11]. For example, in bimodal force microscopy [6–8,12], two modulation signals resonantly drive the cantilever at two eigenmodes simultaneously. In the amplitude modulation mode, two lock-in amplifiers demodulate the signal with respect to both driving frequencies. Thus, one obtains the amplitude and phase signal for both oscillations. The fundamental eigenmode provides the amplitude signal for topography feedback, whereas the amplitude and phase of the higher eigenmode encode the material contrast.

According to previous experiments and theoretical simulations, the second eigenmode of a cantilever is very sensitive to material variations [6,13]. With standard silicon cantilevers, bimodal force microscopy can enhance material contrast with respect to conventional amplitude-modulation modes [7–8,14–16], with piconewton force sensitivity. Local variations of the Hamaker constant cause material contrast in the attractive imaging regime [8,15]. Repulsive bimodal force microscopy imaging has been demonstrated on graphite and DNA [7] and has been combined with nanotomography for the analysis of semicrystalline polypropylene [14]. The additional oscillation of a higher flexural eigenmode adds compositional information to the signal. Polymers are usually characterized in the repulsive regime [17]. Because the interaction forces between the tip and the sample in repulsive imaging are usually larger than in attractive imaging [18], the additional oscillation needs to be optimized to provide compositional mapping while avoiding interference with the topographic imaging process that may arise due to the nonlinear interaction [19]. Thus, we investigated the relevance of the experimental parameters, such as oscillation amplitudes and setpoint, for an atomic force microscope operating in the bimodal mode.

## Experimental

### Amplitude-and-phase-versus-distance curves

We performed amplitude-and-phase-versus-distance (APD) measurements on both freshly cleaned silicon and polystyrene (nominal Young’s modulus of 2.7 GPa; test sample from Bruker AFM Probes, Camarillo, CA) using a Cypher AFM (Asylum Research, Santa Barbara, CA). All of the components required for bimodal operation were implemented in the instrument by the manufacturer. We concurrently recorded the amplitude of the first and second eigenmodes (*A*_1_, *A*_2_), as well as the phase shifts (Δ
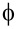
_1_, Δ
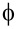
_2_) between the cantilever oscillation and excitation. Following [18], we use the following phase convention: A phase shift Δ
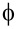
_1_ between the cantilever oscillation and the driving signal that is larger than 90° indicates a net attractive regime, in which van der Waals forces dominate the interaction. Smaller values indicate a net repulsive regime, in which Pauli repulsion becomes increasingly dominant.

The ratio of the amplitudes is crucial for the contrast in the bimodal mode [8,14,19]. In the following, we refer to the ratio *A*_01_/*A*_02_ of the free oscillation amplitudes of the cantilever. The amplitudes were calibrated by advancing the vibrating cantilever toward a silicon surface and recording the amplitude signal (in volts) versus the tip–sample separation (in nanometers) for each mode individually. A linear approximation of the functional dependence of the amplitude on the tip–sample separation yielded the photodiode sensitivity under the assumption that the tip did not indent the silicon sample surface. The free amplitude of the first eigenmode is another crucial parameter because this choice determines the interaction regime. Relatively small free amplitudes (typically, *A*_01_ < 15 nm) allow one to keep the cantilever oscillating in the net attractive regime for all *z*-distances. Larger free amplitudes (typically, *A*_01_ > 40 nm) imply a quick transition from the net attractive to the net repulsive regime upon lowering of the amplitude setpoint. Other parameters such as the cantilever or sample stiffness may further affect the transition. We identified a range of free amplitudes of *A*_01_ = 20–27 nm as the relevant imaging conditions for the following experiments. Thus, a stable repulsive regime could be achieved while excessive tip–sample forces were avoided.

### Bimodal imaging

**Figure 1:**
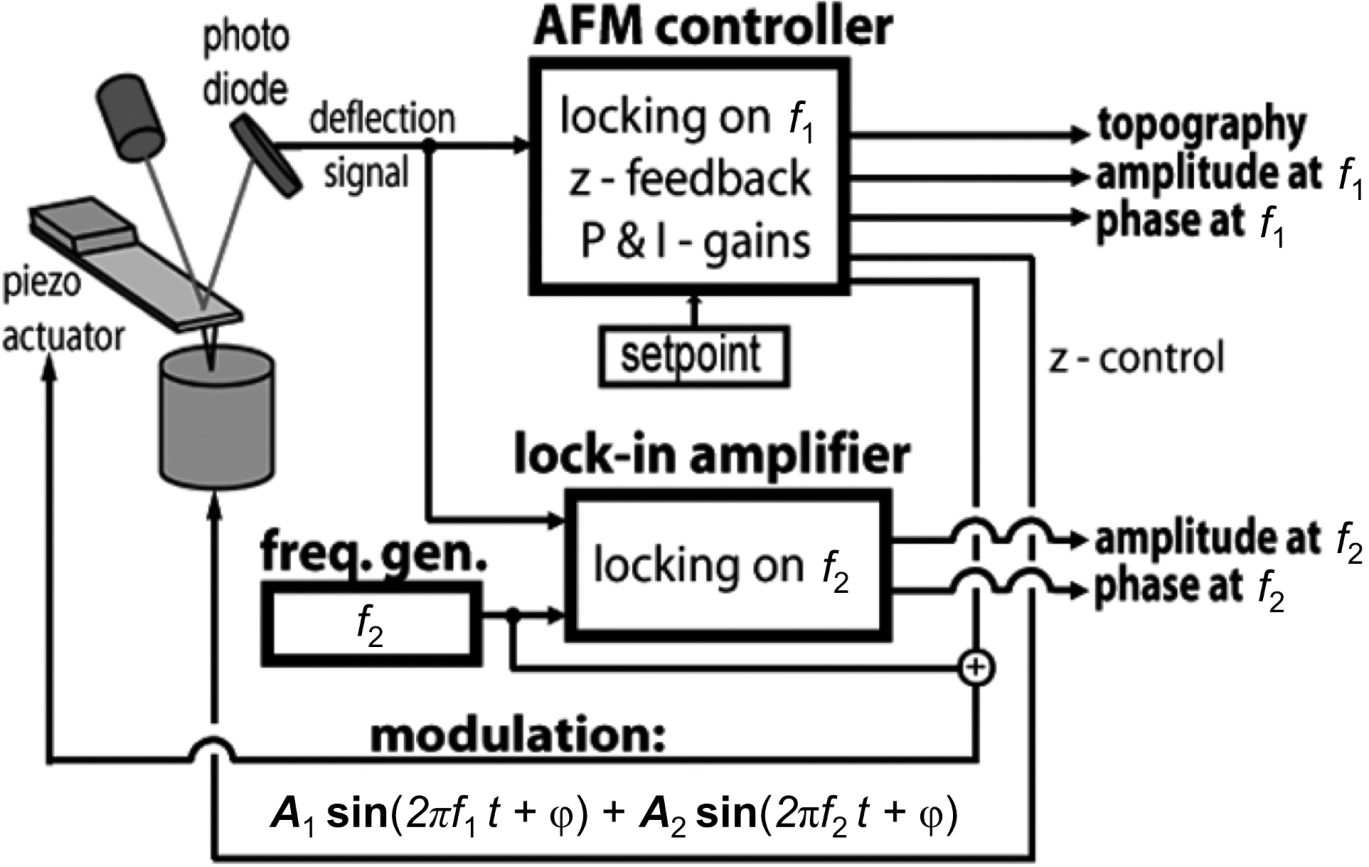
In a bimodal AFM setup, two eigenmodes are driven simultaneously using the same dither piezo. Correspondingly, two lock-in amplifiers (one external and one inside the AFM controller) are used to analyze the deflection signals recorded by the segmented photodiode.

The imaging of a thin film of a polystyrene-*block*-polybutadiene (SB) diblock copolymer was conducted on a Dimension 3100 AFM with a Nanoscope IV controller (Veeco Metrology Inc., Santa Barbara, CA) extended with an external setup for bimodal AFM [7–8]. The system was equipped with a Signal Access Module and special circuitry to access the deflection signals directly at the segmented photodiode, as shown in Figure 1. A digital function generator (33220A; Agilent Technologies Inc., Santa Clara, CA) was used to drive the second eigenmode. The second eigenmode response was measured with a lock-in amplifier (SR-844; Stanford Research Systems Inc., Sunnyvale, CA) set to a time-constant of 100 µs and a filter slope of 6 dB/octave. We used silicon cantilevers (NanoAndMore GmbH, Wetzlar, Germany) with a nominal fundamental resonance of 130 kHz and a nominal flexural stiffness of 30 N/m.

A 60 nm thick film of SB diblock copolymer (*M*_W(PS)_ = 13600 g/mol; *M*_W(PB)_ = 33700 g/mol) was prepared as a test specimen for bimodal AFM. SB is a diblock copolymer with a polydispersity of *M*_w_/*M*_n_ = 1.03 (Polymer Source Inc., Montreal, Canada). The polymer was dissolved in toluene (1 wt %) and spin coated (1500 min^−1^) onto a polished silicon(100) substrate, which was cleaned with ethanol and acetone by ultrasonic treatment for 10 min each. After the evaporation of the toluene and annealing at a fixed vapor pressure of chloroform for several hours, polystyrene microdomains formed a layer of perforated lamellae or cylinders oriented either perpendicularly or in parallel to the sample surface, and these cylinders are surrounded by polybutadiene [20–21]. At room temperature, the polystyrene block is stiffer than the highly compliant polybutadiene within the diblock copolymer because of differences in their glass-transition temperature [22].

## Results and Discussion

### Amplitude-and-phase-versus-distance curves

APD curves provide insight into both the dynamics and the nature of the interactions of the vibrating tip with the sample [23]. To characterize the interaction regime, three types of APD curves were obtained. Two monomodal curves, one each for the first and second eigenmodes, and a bimodal APD curve were obtained for two specimens, silicon and a polystyrene film. In monomodal operation, the repulsive interaction between the tip and the sample is short (typically less than 10% of a cycle) and has a sharply peaked repulsive force and adhesion caused by a water meniscus [24]. The interaction peak for polystyrene is broader, and energy loss is caused by viscous damping [25]. Comparing the bimodal APD curves of both materials can thus help to identify features that are characteristic of polymers.

**Figure 2:**
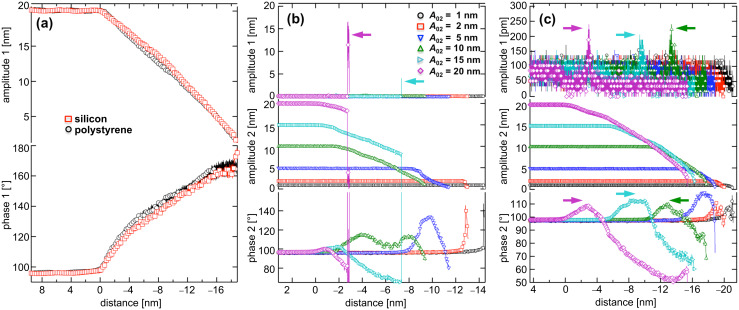
(a) Monomodal APD curves obtained for silicon (red squares) and polystyrene (black circles) by exciting the first eigenmode (free amplitude *A*_01_ = 20 nm). (b) Monomodal APD curves taken for silicon by exciting the second eigenmode of the cantilever to various free amplitudes (*A*_02_ = 1 nm, 2 nm, 5 nm, 10 nm, 15 nm and 20 nm). (c) Similar curves measured for polystyrene. For better visibility, all curves are shifted.

The driving amplitude for the fundamental mode was set to obtain the net attractive regime in monomodal operation. Figure 2a shows APD curves obtained for silicon (red squares) and polystyrene (black circles). The distance is the separation between the undeflected tip and the sample surface. A series of ten APD curves was captured to ensure reproducibility. The curves measured for silicon as well as for polystyrene showed a similar shape. At a given point during the approach, the freely vibrating cantilever began interacting with the sample surface under the influence of attractive forces. We defined the *z*-distance to be zero at this point in all APD diagrams for the leftmost curve; all curves were shifted for better visibility. In addition, the amplitude decreased approximately linearly with approach distance until the tip finally stuck to the surface. The phase shift Δ
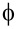
_1_ between the cantilever oscillation and excitation varied from 90°, initially, to 160°. There was no transition to the net repulsive regime (phase shift Δ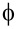 < 90°) under the chosen parameters (free oscillation amplitude *A*_01_ = 20 nm) even for small tip–sample separations. This prevented the tip from indenting into the polymer and led to coincident slopes for both of the amplitude-versus-distance curves.

The APD curves for a monomodal excitation of the second eigenmode for various free amplitudes (1 nm ≤ *A*_02_ ≤ 20 nm; see legend) measured for silicon can be seen in Figure 2b. For small oscillation amplitudes (*A*_02_ = 1 and 2 nm), the oscillating cantilever remained in the net attractive regime for all *z*-distances. In the case of higher oscillation amplitudes, a transition to the repulsive interaction regime was observed after an approach of several nanometers. An interesting effect occurred for amplitudes *A*_02_ ≥ 15 nm. The amplitude abruptly decreased at values smaller than 7 nm (*A*_02_ = 15 nm) or 3 nm (*A*_02_ = 20 nm). At the same *z*-distance, the first eigenmode was excited (indicated by the arrows in the topmost graph). Because both modes are slightly coupled, energy transfer between them can occur [26]. The amplitude of the first eigenmode, indirectly excited through the coupling between the two modes, nearly reaches the initial amplitude of the second eigenmode at the same *z*-distance before decreasing. No further approach data could be acquired because the trigger value, i.e., the target amplitude for the approach, was reached. The amplitude and phase behavior measured for the polystyrene sample is similar to that for silicon (Figure 2c). The transition between the net attractive and net repulsive regimes was rather smooth and without an abrupt drop of the second-mode amplitude. Nevertheless, for higher amplitudes (*A*_02_ ≥ 10 nm), coupling between the two modes was observed at the exact distance where the net attractive forces between the cantilever and the polystyrene reached their peak level (see arrows). A first-eigenmode amplitude of 200 pm was detected although the first mode was not driven by the shaker piezo. However, this small oscillation, which was close to the detection limit of the instrument, did not affect the oscillation of the second eigenmode.

**Figure 3:**
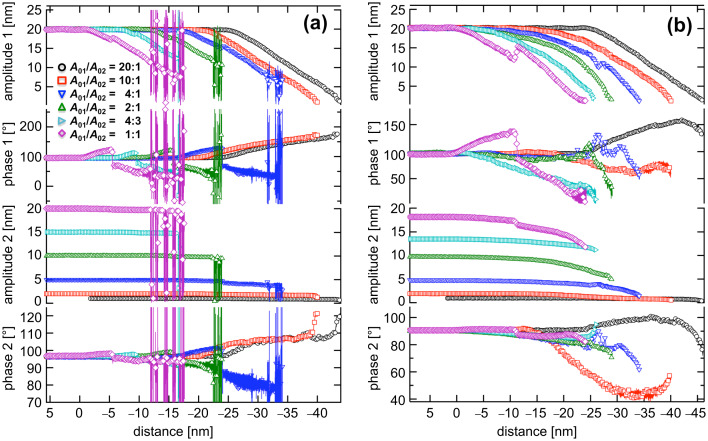
Bimodal APD curves for (a) silicon and (b) polystyrene (*A*_01_ = 20 nm) obtained with simultaneous excitation of the first two fundamental eigenmodes (*f*_1_ = 122 kHz, *f*_2_ = 785 kHz) for various amplitude ratios: *A*_01_ /*A*_02_ = 20:1, 10:1, 4:1, 2:1, 4:3, and 1:1.

We switched from monomodal to bimodal excitation and performed similar experiments on silicon (Figure 3a) and polystyrene (Figure 3b). The same amplitude values were used, implying the ratios *A*_01_/*A*_02_ = 20:1, 10:1, 4:1, 2:1, 4:3 and 1:1. For large ratios (*A*_01_/*A*_02_ = 20:1 and 10:1), the system stayed in a state of net attractive interaction during the entire approach. The oscillation of the fundamental eigenmode was hardly affected by the additional oscillation. Increasing the amplitude of the second eigenmode to 5 nm (*A*_01_/*A*_02_ = 4:1) and greater led to a transition to net repulsive forces in the approach curve at 5–7 nm after the first interaction between the tip and the sample occurred. Such a stabilization of the repulsive regime has been previously observed [27]. During closer approaches, the amplitude of the higher eigenmode is only slightly influenced despite increasing interactions. Crosstalk between the two modes occurred for a *z*-distance of approximately 10 to 14 nm, at which the amplitude *A*_1_ decreased whereas the second eigenmode was enhanced. For lower ratios, the same effect was observed even more distinctly (see the diamond symbols in Figure 3a). For polystyrene (Figure 3b), all the curves reveal a transition from the net attractive region to a net repulsive region in Δ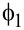 except for the curve with a second-mode free amplitude of *A*_02_ = 1 nm. Interestingly, in that case, only the second-eigenmode oscillation makes the transition to a net repulsive force. No crosstalk was found during the entire approach for all amplitude ratios.

The results show that, for large amplitude ratios and within the accuracy of the measurement, the additional oscillation of the second eigenmode weakly affects the shape of the first-eigenmode amplitude curve. The higher-mode oscillation is an additional oscillation to the fundamental oscillation. The instantaneous amplitude of the combined motion varies: *A*_01_ − *A*_02_ ≤ *A*_inst_ ≤ *A*_01_ + *A*_02_. Under such conditions, the higher-frequency oscillation only slightly affects topographical feedback because the first eigenmode is barely affected by the second eigenmode. The response of the second-eigenmode amplitude, however, was strongly influenced by the oscillation of the fundamental eigenmode. Nevertheless, a high amplitude setpoint ratio *A*_1_/*A*_01_ for topographical feedback should be chosen; otherwise, crosstalk can occur. Imaging with small amplitude ratios *A*_01_/*A*_02_ on stiff samples (e.g., silicon) is not stable due to a strong crosstalk between the two modes under the influence of repulsive forces. Chaotic cantilever motion has been predicted for such conditions [19].

For softer samples such as polymers, smaller amplitude ratios can be used, allowing stronger repulsive interactions between the tip and the sample to enhance the contrast in the phase images. An operation regime was found at very low setpoint ratios in which the oscillation of the first eigenmode apparently indicates a net attractive regime whereas the higher eigenmode indicates a change in the interaction. This observation may be useful in establishing a method to separate attractive and repulsive contributions to the interaction force. To this end, it has to be proven whether such low setpoint ratios lead to stable imaging conditions. Bimodal APD curves may also give further insight into the various modes of energy dissipation because bimodal APD curves depict such distinctive shapes. Further experiments and simulations will lead to a better understanding of the complex tip–sample dynamics in repulsive bimodal operations.

### Imaging of a polystyrene-*block*-polybutadiene diblock copolymer

**Figure 4:**
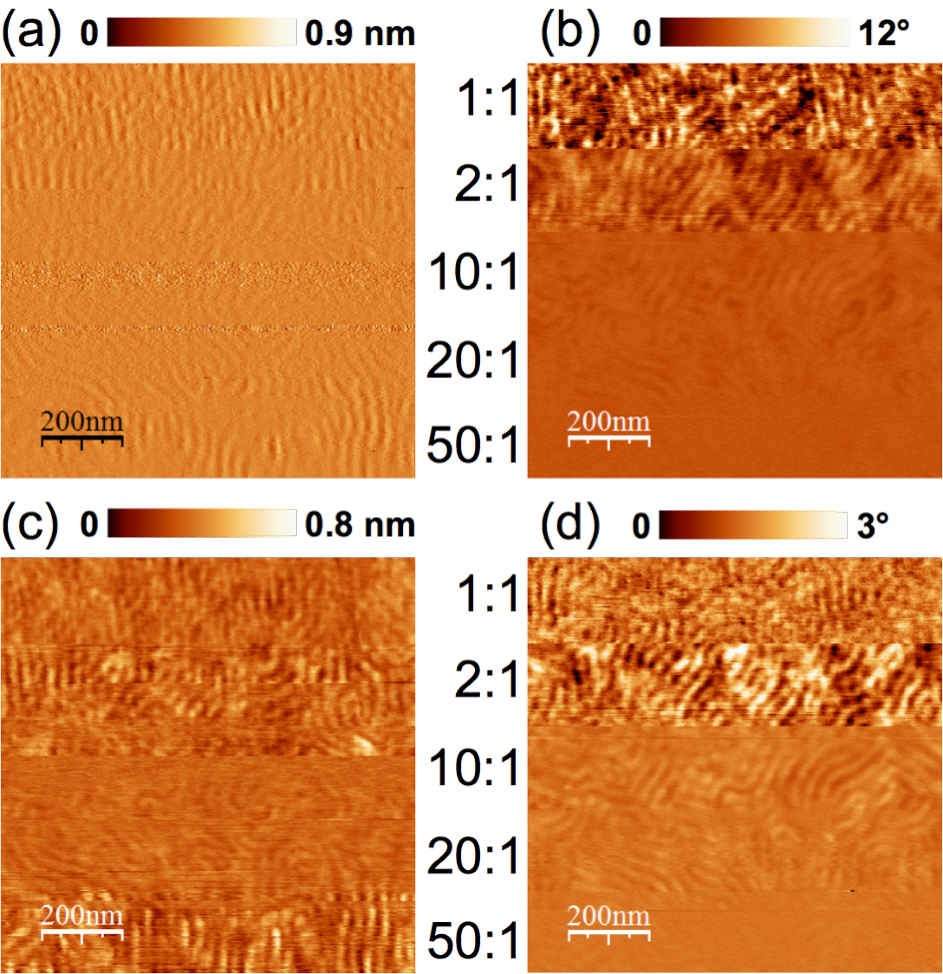
First (a,b) and second eigenmode (c,d) amplitude (a,c) and phase images (b,d) measured on the surface of a thin film of a cylinder forming SB diblock copolymer. The amplitude ratio *A*_1_/*A*_2_ was varied from 50:1 to 1:1 while keeping *A*_01_ = 27 nm constant. The best contrast was observed for ratios between 10:1 and 2:1.

We explored the imaging capabilities of repulsive bimodal AFM with a 60 nm thick film of polystyrene-*block*-polybutadiene (SB) diblock copolymer. To this end, we systematically varied the amplitude ratios *A*_01_/*A*_02_ between the first and second eigenmodes for bimodal imaging while keeping *A*_01_ = 27 nm constant. Figure 4 shows the recorded amplitude (a) and phase images (b) of the first eigenmode as well as the amplitude (c) and phase images (d) of the second eigenmode while varying the amplitude ratio *A*_01_/*A*_02_ between the two eigenmodes stepwise from 1:1 (top) to 50:1 (bottom). The amplitude of the first eigenmode did not change considerably because the feedback of the instrument kept this parameter constant. The two polymer blocks of the cylindrical structure of the block copolymer are increasingly indistinguishable in the phase image of the first eigenmode (Figure 4b) when increasing the amplitude ratio. By contrast, there is an optimum amplitude ratio with respect to the contrast in the second eigenmode images. We find that the best contrast is obtained for amplitude ratios smaller than 10:1, which is different from the results obtained in the attractive regime, where the optimum contrast implies free-amplitude ratios larger than 10:1 [28]. Note that the image contrast is related to the signal-to-noise ratio and thus difficult to quantify for heterogeneous samples. The conclusions drawn here are on the basis of the optical impression of the authors.

Comparing these observations with the bimodal-spectroscopy measurements on polystyrene from the previous section (Figure 3b), leads to the same conclusions. The maximum phase shift Δ
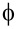
_1_ between the oscillator at resonance (far away from the sample surface) and at the closest tip–sample distance (lowest amplitude) has its highest value for an amplitude ratio of 1:1 and decreases with increasing ratio. This agrees with Figure 4b. The total free amplitude *A*_01_+*A*_02_ is highest for an amplitude ratio of 1:1 and leads to the highest impact of the tip on the sample and hence to the maximum phase response. In the case of the second eigenmode and considering only the repulsive regime, the maximum phase response ratio *A*_01_/*A*_02_ is between 10:1 and 4:1 at a given amplitude (setpoint), which is in good agreement with the contrast found in Figure 4d. When operating the AFM in the repulsive regime, we assume that there is a minimum amplitude of the second eigenmode necessary to sense the mechanical differences between both types of polymers, which differ considerably in stiffness. However, if the amplitude of the additional oscillation becomes too large, the bimodal technique may become destructive to the surface structure, counteracting a good phase contrast.

**Figure 5:**
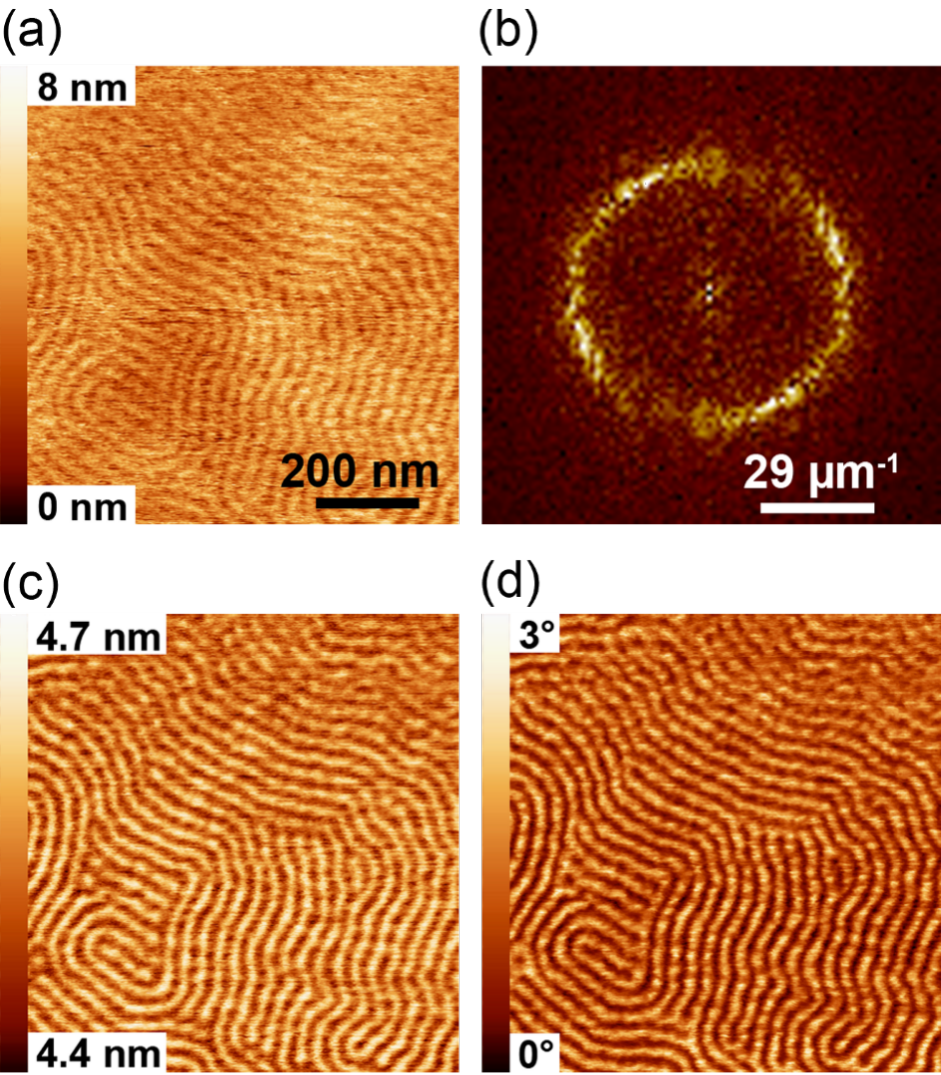
Bimodal AFM images measured on the surface of a thin film of a cylinder formed of SB diblock copolymer: (a) topography, (c) second-eigenmode amplitude and (d) phase shift. (b) The Fourier transform was calculated from the material contrast (d).

Figure 5 shows an SB sample measured in the repulsive regime for a bimodal amplitude ratio of *A*_01_/*A*_02_ = 6.6:1. We worked at a setpoint amplitude of 90% (29.6 nm at 33 nm free amplitude A_01_) of the first eigenmode. The topographic data are shown in Figure 5a. Images obtained from the first eigenmode under bimodal operation are comparable to data from conventional single-mode amplitude-modulation imaging (e.g., [22]; data not shown). We recorded amplitude and phase images for the second eigenmode. The amplitude signal of the second eigenmode (Figure 5c) reveals local mechanical and dissipative properties of the thin film. For PS, the oscillation amplitude of the second eigenmode is smaller (4.5 nm) than for PB (4.7 nm). The average damping compared to the free amplitude is 90% and 95% for the PS and PB parts of the SB sample, respectively. This means that the PB part is more compliant and more dissipative than the PS part, which has already been reported for the same sample based on the results of a resonant shear force experiment [29]. Thus, when imaging flat samples that present such varying elastic properties, topographic contrast is enhanced by the repulsive-imaging process. At a given setpoint, the softer component is substantially deformed, whereas the stiffer material remains unaffected. Thus, stiff materials always appear as elevated features with respect to compliant regions, when imaging in the repulsive regime [22,30]. The phase image (Figure 5d) also showed a clear contrast between the blocks of the copolymer. From this compositional contrast, we calculated a two-dimensional Fourier transformation as presented in Figure 5b with a maximum at a spatial frequency of 29 µm^−1^. This frequency corresponds to a pitch between the cylinders of polystyrene of 33.5 nm.

**Figure 6:**
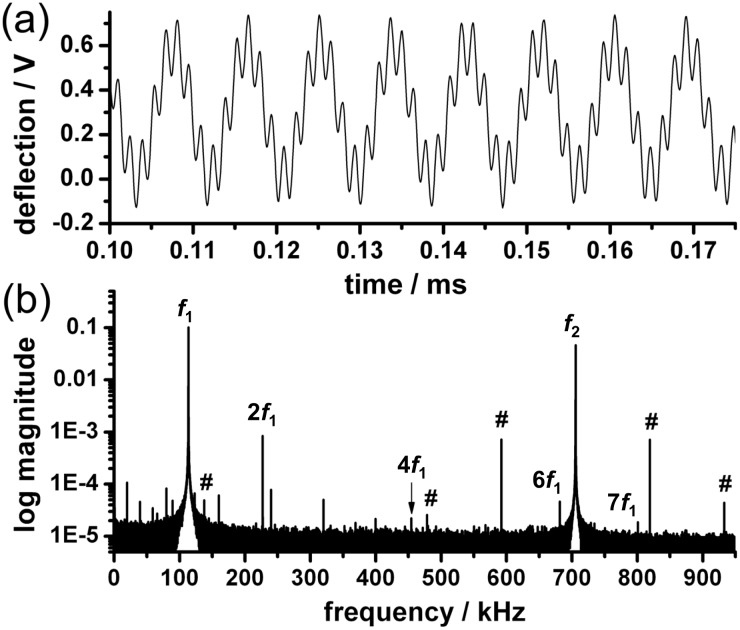
(a) Bimodal AFM deflection signal and (b) Fourier analysis of the time trace obtained for the SB sample surface. (a) In the time trace, the oscillations are sinusoidal at both eigenmodes. (b) The Fourier transform reveals mechanical mixing between the eigenmodes. Sidebands of the second eigenmode are marked by “#”. Several harmonics (*f*_1_, 2*f*_1_, 4*f*_1_, 6*f*_1_, and 7*f*_1_) of the fundamental eigenmode can be observed above the noise level.

Intermodulation effects can occur when an oscillating nonlinear system is driven at two distinct frequencies. This effect can be used to measure mechanical sample properties with an AFM [9,31]. In bimodal force microscopy, a spectral analysis of the system response allows one to distinguish between stable (quasi-) periodic and chaotic regimes. The time series shown in Figure 6a makes clear that the cantilever response under bimodal operation was sinusoidal in both the first (*f*_1_ = 113.5 kHz) and second eigenmodes (*f*_2_ = 705.6 kHz). Furthermore, a Fourier analysis of the time traces (Figure 6b) helps to assess the signal-to-noise ratio of the signals. For the first eigenmode, we find a ratio of 10^4^, and for the second eigenmode, approximately 5 × 10^3^. Integer harmonics of the fundamental eigenmode (*f*_1_ = 113.5 kHz) prevail above the noise level at 2*f*_1_ = 227.1 kHz, 4*f*_1_ = 454.2 kHz, 6*f*_1_ = 681.3 kHz, and 7*f*_1_ = 801.0 kHz. For the second eigenmode, we find frequency mixing with the lower eigenmode, resulting in symmetric sidebands. This results in the peaks *f*_2_ + *f*_1_ = 819.1 kHz and *f*_2_ – *f*_1_ = 592.0 kHz for direct mixing between the first and second eigenmodes and *f*_2_ + 2*f*_1_= 932.7 kHz and *f*_2_ – 2*f*_1_ = 478.4 kHz for the first eigenmode mixing with the second harmonic of the first eigenmode. We can even observe the *f*_2_ – 5*f*_1_ = 137.7 kHz peak for mixing with the fifth harmonic of the first eigenmode. However, the higher harmonic oscillations and the sidebands due to frequency mixing between the eigenmodes were smaller than the signals of *f*_1_ and *f*_2_ by at least two orders of magnitude. Thus, we conclude that stable imaging with only minimal nonlinear effects is possible for gentle imaging conditions in the repulsive regime.

## Conclusion

Bimodal AFM imaging is fully compatible with repulsive operation. We found that the first-eigenmode image quality in the repulsive regime is not affected by the second-eigenmode excitation for large amplitude ratios, *A*_01_/*A*_02_. For small ratios, a crosstalk between the two eigenmodes occurred for stiff samples (e.g., silicon), rendering a stable operation of the AFM impossible. On softer samples (e.g., polystyrene), operational parameters corresponding to different operation regimes for both modes were found. To optimize the imaging of heterogeneous polymers, the amplitude ratio is a key parameter. On the SB sample, the optimum amplitude ratio for imaging polymer samples in the repulsive regime was less than 10:1. Our data imply that the small oscillation of the second eigenmode does not affect the amplitude behavior of the first eigenmode at setpoint ratios that are typically used for imaging. Under such conditions, the imaging process seems to be largely independent of the additional modulation. A stable repulsive regime is also indicated by the time-trace analysis, which shows regular oscillations in both eigenmodes with only minimal nonlinear effects.

We would like to emphasize that the results shown here were obtained on silicon and polystyrene samples, as well as on an SB block copolymer, using a particular type of cantilever: i.e., at a fixed ratio between the cantilever stiffness and the effective tip–sample stiffness. It will be interesting to explore the impact of stiffness further. From an experimental point of view, such an experiment is demanding, as a very large detection bandwidth is needed. By contrast, using short, soft cantilevers may allow the mechanical characterization of biomolecules.
